# Image distortion correction for MRI in low field permanent magnet systems with strong *B*_*0*_ inhomogeneity and gradient field nonlinearities

**DOI:** 10.1007/s10334-021-00907-2

**Published:** 2021-01-27

**Authors:** Kirsten Koolstra, Thomas O’Reilly, Peter Börnert, Andrew Webb

**Affiliations:** 1 Radiology, Division of Image Processing, Leiden University Medical Center, Albinusdreef 2, 2333 ZA Leiden, The Netherlands; 2 Radiology, C.J. Gorter Center for High-Field MRI, Leiden University Medical Center, Albinusdreef 2, 2333 ZA Leiden, The Netherlands; 3 Philips Research, Röntgenstraβe 24-26, 22335 Hamburg, Germany

**Keywords:** Distortion correction, B_0_ mapping, Conjugate phase reconstruction, Model-based reconstruction, Low field MRI

## Abstract

**Objective:**

To correct for image distortions produced by standard Fourier reconstruction techniques on low field permanent magnet MRI systems with strong ${B}_{0}$ inhomogeneity and gradient field nonlinearities.

**Materials and methods:**

Conventional image distortion correction algorithms require accurate ${\Delta B}_{0}$ maps which are not possible to acquire directly when the ${B}_{0}$ inhomogeneities also produce significant image distortions. Here we use a readout gradient time-shift in a TSE sequence to encode the ${B}_{0}$ field inhomogeneities in the k-space signals. Using a non-shifted and a shifted acquisition as input, $\Delta {B}_{0}$ maps and images were reconstructed in an iterative manner. In each iteration, $\Delta {B}_{0}$ maps were reconstructed from the phase difference using Tikhonov regularization, while images were reconstructed using either conjugate phase reconstruction (CPR) or model-based (MB) image reconstruction, taking the reconstructed field map into account. MB reconstructions were, furthermore, combined with compressed sensing (CS) to show the flexibility of this approach towards undersampling. These methods were compared to the standard fast Fourier transform (FFT) image reconstruction approach in simulations and measurements. Distortions due to gradient nonlinearities were corrected in CPR and MB using simulated gradient maps.

**Results:**

Simulation results show that for moderate field inhomogeneities and gradient nonlinearities, $\Delta {B}_{0}$ maps and images reconstructed using iterative CPR result in comparable quality to that for iterative MB reconstructions. However, for stronger inhomogeneities, iterative MB reconstruction outperforms iterative CPR in terms of signal intensity correction. Combining MB with CS, similar image and $\Delta {B}_{0}$ map quality can be obtained without a scan time penalty. These findings were confirmed by experimental results.

**Discussion:**

In case of ${B}_{0}$ inhomogeneities in the order of kHz, iterative MB reconstructions can help to improve both image quality and $\Delta {B}_{0}$ map estimation.

**Supplementary Information:**

The online version contains supplementary material available at 10.1007/s10334-021-00907-2.

## Introduction

Low field permanent magnet MRI systems based on cylindrical Halbach arrays typically have relatively large ${B}_{0}$ inhomogeneities (hundreds to thousands of parts per million (ppm)) due to the finite diameter-to-length ratio. Additional inhomogeneities are introduced by small variations in the properties of the discrete elements of magnetic material used in these systems as well as by manufacturing tolerances in the construction of the system as a whole [1–6]. Such inhomogeneities lead to image distortions when using fast Fourier transform (FFT)-based reconstructions. A large number of different methods have been proposed to correct for these type of distortions, which are typically most prevalent at clinical field strengths when using echo-planar imaging (EPI) readouts [7–12]. All these methods either require long scanning times when applied to spin-echo type acquisitions, needed at low field for their comparatively low sensitivity to field inhomogeneities, or rely on having an accurate ${B}_{0}$ map. At low field, model-based (MB) image reconstructions have also been proposed to take into account nonlinear encoding fields in the reconstruction process, although in a basic framework which again requires accurate field map information [4, 13]. Standard ${B}_{0}$ mapping techniques assume that the phase in a pixel of the image is given by the phase of the sampled signal at the echo-top. This allows the estimation of $\Delta {B}_{0}$ from the phase difference map corresponding to two images acquired with different echo times in a gradient echo sequence [14]. While fat complicates ${B}_{0}$ mapping at high field, at low field the water-fat shift and hence the phase accumulation for the fat signal compared to the water signal is negligible. However, for very strong inhomogeneities, the assumption that the phase in a pixel is given by the signal phase at the echo top no longer holds and leads to inaccurate ${\Delta B}_{0}$ maps. Therefore, for the case of strongly inhomogeneous ${B}_{0}$ fields, there is a need for a modified ${B}_{0}$ mapping approach that first performs a phase correction for the individual source images, taking into account the strong inhomogeneities in the ${B}_{0}$ field. For low field MRI, this approach must also be applicable to measurements with relatively low SNR.

This work presents a joint image reconstruction and ${B}_{0}$ mapping scheme in which ${B}_{0}$-induced phase changes during sampling are corrected for by two types of image reconstruction: conjugate phase reconstruction [15, 16] (CPR) and MB reconstruction [4, 17]. This is done in an iterative framework such that the algorithm does not require an estimate of the ${B}_{0}$ field as input. In systems with severe ${B}_{0}$ inhomogeneities which produce spectral linewidths on the order of hundreds of Hz, conventional gradient-echo imaging is not feasible due to rapid dephasing of the MR signal. Therefore, ${B}_{0}$ encoding was obtained by using a time-shifted turbo spin echo (TSE) sequence. The proposed reconstruction techniques are compared to the standard fast Fourier transform, which is typically used to reconstruct the source images for a $\Delta {B}_{0}$ map estimation. Since ${B}_{0}$ mapping requires two acquisitions with the TSE sequence, one without the time-shift and one with, the total imaging time is doubled compared with a single image acquisition. Therefore, we furthermore investigated the combination of MB reconstructions with compressed sensing (CS) using an undersampling factor of 2, to demonstrate that the ${B}_{0}$ information can be encoded in this single-coil setup without penalties on the total scan time. Correction for image distortion due to spatial gradient nonlinearity patterns was finally integrated in both CPR and MB approaches. The accuracy of the images and the $\Delta {B}_{0}$ maps is quantified in a simulation study, and the feasibility of these techniques is demonstrated for phantom and in vivo measurement data.

## Materials and methods

### Experimental setup

**Fig. 1:**
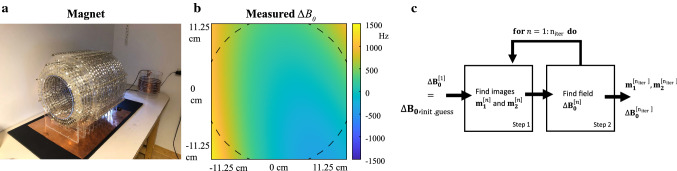
Magnet setup. **a** The Halbach magnet, producing an 0.05 T magnetic field at the center. **b** The ${B}_{0}$ field was measured on a 225 × 225 × 300 mm^3^ with a gaussmeter connected to a robot. After subtraction of the center frequency and conversion to Hz it was fitted to a basis of second-order spherical harmonics. The field in the center transversal slice of the magnet shows inhomogeneities up to approximately ± 1200 Hz. The dashed lines indicate the position of the inner diameter of the magnet. **c** Schematic overview of the reconstruction algorithm for iterative CPR and iterative MB. In each iteration, two images are reconstructed from the unshifted and shifted acquisitions. The phase of the resulting images is used to map the $\Delta {B}_{0}$ field. The $\Delta {B}_{0}$ map is used as input in the CPR and MB reconstruction in the next iteration

The low field system is a *k* = 1 Halbach magnet, constructed as described in [18], producing an 0.05 T (2.15 MHz) magnetic field at the center of the bore. The main magnetic field was measured in a 225 × 225 × 300 mm^3^ field of view (FOV) at 5 × 5 × 5 mm^3^ resolution using a gaussmeter (Lake Shore Cryotronics, Westerville, OH) connected to a 3D positioning robot [19]. The center frequency was subtracted from the field map, which was subsequently converted to Hz and fitted to a basis of spherical harmonics up to 15th order ($\frac{{\Vert {B}_{0,\text{fitted}}-{B}_{0,\text{measured}}\Vert }_{2}}{{\Vert {B}_{0,\text{measured}}\Vert }_{2}}$ = 10^–5^) to remove measurement noise. This map was used as the input for the simulations. A single RF solenoid was used for signal transmission and reception: an elliptical spiral solenoid [20] (inner diameter 1/inner diameter 2/length = 234/194/150 mm) was used for brain scans and a cylindrical solenoid (inner diameter/length = 150/150 mm) was used for phantom scans. The magnet and the measured main field, converted to Hz, are shown in Fig. 1a, b, respectively. ${B}_{0}$ and $\Delta {B}_{0}$maps are given in Hz throughout the paper.

### MR data simulation

To compare the performance of different reconstruction approaches, 2D k-space data was simulated for a 2D MATLAB Shepp–Logan phantom using the signal model

1 $$y\left(\mathbf{k}(t)\right)= \int m\left(\mathbf{x}\right){e}^{-2\pi \Delta {B}_{0}\left(\mathbf{x}\right)i(t+{t}_{\mathrm{shift}})}{e}^{-\mathbf{k}(t)\cdot \mathbf{x}i}d\mathbf{x},$$

where $m$ is the spin density, ${\Delta B}_{0}$ is the measured field deviation converted to Hz, ${t}_{\mathrm{shift}}$ is the time-shift in the readout gradient in seconds, $y$ is the time domain signal for k-space location $\mathbf{k}$ at time *t*. Any spatial nonlinearities in the frequency and phase encoding gradients were ignored in this process if not mentioned otherwise. Simulations were performed with a readout bandwidth (BW) of 20 kHz, a FOV of 225 × 225 mm^2^ and a time-shift for the readout gradient (${t}_{\mathrm{shift}}$) of 100 µs, which is short enough to prevent phase wrapping. Time-domain signals were always generated on a four times larger grid than the reconstruction grid to simulate multiple frequencies within each voxel. Note that for reconstruction the number of voxels in the ${\Delta B}_{0}$ map was set to match the number of voxels in the reconstructed image, 128 × 128, resulting in a resolution of 1.75 × 1.75 mm^2^ and a pixel BW of 156 Hz/pixel. The number of phase and frequency encoding steps was set to 128. These settings correspond to a water-fat shift of 1/20 pixel. White Gaussian noise was added to the simulated complex time-domain signals such that the resulting SNR was approximately 20. These simulations were repeated for two different transverse slices: in the center and 7.5 cm off-center in the foot-head direction.

### MR data acquisition

MR data were acquired in one healthy volunteer in vivo and for two different phantoms. For each case, two 3D TSE k-space data sets with an unshifted and shifted readout gradient were acquired using a center-out phase-encoding direction. The following acquisition parameters were used:

In vivo brain: FOV = 224 × 224 × 200 mm^3^, TE = 20 ms, TR = 500 ms, ETL = 4, pixel BW = 156 Hz/pixel, resolution = 1.75 × 1.75 × 4.0 mm^3^, RF pulse length = 100 µs, ${t}_{\mathrm{shift}}$ = 150 µs, scan time: 10 min per acquisition (one with the time-shift and one without). The 20% of k-space outside a cylindrical mask was not acquired to reduce scan time.

45 Equally spaced tubes filled with sunflower oil: FOV = 192 × 192 × 60 mm^3^, TE = 20 ms, TR = 500 ms, ETL = 2, pixel BW = 156 Hz/pixel, resolution = 1.5 × 1.5 × 5.0 mm^3^, RF pulse length = 100 µs, ${t}_{\mathrm{shift}}$ = 50 µs, scan time: 6:24 min per acquisition (one with the time-shift and one without).

The phantom data was used to investigate how well the algorithms perform when the main magnetic field was deliberately deshimmed by having a constant current running through one of the gradient coils. In a similar vein, the in vivo brain scan was furthermore acquired a second time using an external magnet attached to the Faraday cage, to increase the ${B}_{0}$ inhomogeneities in such a way that the field in the center slice resembled that of the 7.5 cm off-center slice used for simulations. In each of the experiments, the time-shift of the readout gradient was set according to the level of expected field inhomogeneity in the imaging FOV, such that phase wraps did not occur.

To test ${B}_{0}$ correction combined with gradient nonlinearity correction, the following data set was acquired in coronal orientation, in which the gradient nonlinearity pattern is strong compared to that in transverse orientations.

63 Equally spaced tubes filled with sunflower oil: FOV = 256 × 256 × 100 mm^3^, TE = 18 ms, TR = 500 ms, ETL = 2, pixel BW = 156 Hz/pixel, resolution = 2.0 × 2.0 × 5.0 mm^3^, RF pulse length = 100 µs, ${t}_{\mathrm{shift}}$ = 150 µs, scan time: 10:40 min per acquisition (one with the time-shift and one without).

### Image reconstruction

Reconstruction of the images was performed in MATLAB (Mathworks Inc, Natick, MA) and run on a Windows 64-bit machine with Intel Xeon CPU E5-1620 v3 @3.5 GHz and 32 GB internal memory. All data sets were pre-processed by applying a 3D sine-bell squared filter in the time domain before reconstruction. All reconstructions were performed for a single slice in 2D. To support this, a 3D FFT was first applied to the 3D filtered k-space data to select a slice from the 3D acquisition. To prevent phase wrapping in case of strong static phase shifts ($C$), a phase correction was applied to both images, such that the phase range in the second image is centered around zero. After this, the selected slice was transformed back to the time domain.

From the two 2D k-space data sets, $\Delta {B}_{0}$ maps were reconstructed using three different approaches.

- 1) Image reconstruction using FFT and regularized ${B}_{0}$ mapping based on the phase difference map.
- 2) Set $\Delta {B}_{0}\left(\mathbf{x}\right)=0$ as the initial guess. Iterate: Image reconstruction using FFT and conjugate phase reconstruction (CPR) to correct for ${B}_{0}$-induced phase changes during the readout process [15, 16], ${B}_{0}$ mapping as in 1).
- 3) Set $\Delta {B}_{0}\left(\mathbf{x}\right)=0$ as the initial guess. Iterate: MB image reconstruction taking into account the ${B}_{0}$ field in the encoding matrix [21], ${B}_{0}$ mapping as in 1).

Figure 1c shows a schematic overview of approaches 2 and 3. Reconstructions for approaches 2 and 3 were performed using 3 iterations if not mentioned otherwise.

#### Conjugate phase reconstruction

For CPR, the inverse of signal Eq. (1) was approximated as

2 $$m\left(\mathbf{x}\right)\approx \int y\left(\mathbf{k}(t)\right){e}^{2\pi \Delta {B}_{0}\left(\mathbf{x}\right)i(t+{t}_{\mathrm{shift}})}{e}^{\mathbf{k}(t)\cdot \mathbf{x}i}dt$$

and implemented via multi-frequency interpolation as described in [15], such that fast computation times can be achieved using FFTs. The frequency range was set from −4000 to 4000 Hz and discretized with a step size of 3 Hz for simplicity.

#### Model-based reconstruction

For model-based image reconstruction, signal Eq. (1) was discretized and written into the linear system $E\mathbf{m}=\mathbf{y}$. In this equation, $E\in {\mathbb{C}}^{{N}^{2}\times M}$ is the total encoding matrix in which the encoding matrices for each phase encoding step were stacked, $\mathbf{m}\in {\mathbb{C}}^{M\times 1}$ is the vectorized unknown image and $\mathbf{y}\in {\mathbb{C}}^{{N}^{2}\times 1}$ is a vector containing the time domain signals of length $N$ for all $N$ phase encoding steps. $M$ is the number of voxels in the reconstructed image and is determined by the number of voxels in the initial ${\Delta B}_{0}$ map. In theory, $M$ and ${N}^{2}$ can be different, but in this work, they were set the same to match the resolution of the reconstructed images from the MB approach with those from the FFT and CPR approaches. The corresponding minimization problem was written as

3 $${\hat{\mathbf{m}}} = \mathop {{\text{min}}}\limits_{{\mathbf{m}}} \left\{ {\frac{\mu }{2}||E{\mathbf{m}} - {\mathbf{y}||}_{2}^{2} + \frac{\lambda }{2}\left(|| {\nabla_{x} {\mathbf{m}}||_{1} + ||\nabla_{y} {\mathbf{m}}||_{1} } \right)} \right\}$$

Note that the problem is regularized by enforcing sparsity of the image in the total variation domain, using ${\nabla }_{x},{\nabla }_{y}\in {\mathbb{R}}^{M\times M}$ as first-order backward differential operators. This formulation was solved using the non-linear minimization scheme Split Bregman (SB) [22, 23]. The implementation used 2 outer iterations, 1 inner iteration and a conjugate gradient (CG) tolerance of 10^–2^. The regularization parameters were tuned empirically and set to $\mu =1$ and $\lambda =1$ × 10^–10^ for simulations, $\mu =1$ and $\lambda =$5 × 10^–9^ for measured phantom data and $\mu =1$ and $\lambda =$ 8 × 10^–11^ for measured in vivo data. In the tuning process, the ratio between $\mu$ and $\lambda$ was first determined by analysing the smoothness of the reconstructed images. Second, $\mu$ and $\lambda$ were simultaneously scaled to minimize the amount of residual undersampling artifacts.

#### Model-based reconstruction with compressed sensing

To reduce the total imaging time and to investigate the combination of MB with CS, the k-space data for the brain scan were retrospectively undersampled using a variable density Cartesian undersampling pattern with an undersampling factor of 2 and reconstructed using CS. For this, a diagonal sampling matrix $R\in {\mathbb{R}}^{{N}^{2}\times {N}^{2}}$ was added to the signal model, resulting in the minimization problem

4 $${\hat{\mathbf{m}}} = \mathop {{\text{min}}}\limits_{{\mathbf{m}}} \left\{ {\frac{\mu }{2}||RE{\mathbf{m}} - {\mathbf{y}||}_{2}^{2} + \frac{\lambda }{2}\left( ||{\nabla_{x} {\mathbf{m}||}_{1} + ||\nabla_{y} {\mathbf{m}||}_{1} } \right)} \right\}.$$

For this reconstruction the number of outer SB iterations was set to 3, $\mu =$ 2 × 10^–10^ and $\lambda =$ 2 × 10^–25^ for simulations, $\mu =$ 2 × 10^–4^ and $\lambda =$ 2 × 10^–12^ for phantom measurements and $\mu =$ 2.5 × 10^–4^ and $\lambda =$ 2.5 × 10^–13^ for in vivo measurements.

### *B*_*0*_ mapping

In each iteration, the phase components of the ${B}_{0}$ corrected images were used to map the field according to the signal equation

5 $$\varphi \left( {\mathbf{x}} \right) = - 2\pi t_{{{\text{shift}}}} \Delta B_{0} \left( {\mathbf{x}} \right) + C\left( {\mathbf{x}} \right),$$

with $C\in {\mathbb{R}}^{N\times N}$ a map that accounts for receive phase offsets that are the same for different time shifts ${t}_{\mathrm{shift}}$. Tikhonov regularization was added to obtain smooth $\Delta {B}_{0}$ maps in case of low SNR measurements. This resulted in the final minimization problem

6 $${\hat{\mathbf{b}}} = \mathop {{\text{min}}}\limits_{{\mathbf{b}}} \left\{ ||{A{\mathbf{s}} - {\mathbf{\Phi }||}_{2}^{2} + \frac{\gamma }{2}\left( ||{\nabla_{x} {\mathbf{b}||}_{2}^{2} +|| \nabla_{y} {\mathbf{b}||}_{2}^{2} } \right)} \right\}.$$

In this formulation, $\mathbf{b}\in {\mathbb{R}}^{M\times 1}$ and $\widehat{\mathbf{b}}\in {\mathbb{R}}^{M\times 1}$ are vectorized $\Delta {B}_{0}$ maps, $$\mathbf{s}=\left[\begin{array}{c}\mathbf{b}\\ \mathbf{C}\end{array}\right]$$ and $\mathbf{C}\in {\mathbb{R}}^{M\times 1}$ is the vectorized $C$ map. Furthermore, $A\in {\mathbb{R}}^{2M\times 2M}$ is the system matrix derived from Eq. (5), $$\mathbf{\Phi }=\left[\begin{array}{c}{\mathbf{\Phi }}_{1}\\ {\mathbf{\Phi }}_{2}\end{array}\right]$$ with ${\mathbf{\Phi }}_{\mathrm{i}}\in {\mathbb{R}}^{M\times 1}$ is the vectorized phase map of image number *i.* The regularization parameter was tuned empirically and set to $\gamma$=2 × 10^–10^ for simulations, $\gamma$=2 × 10^–8^ for phantom measurements and $\gamma$=4 × 10^–8^ for in vivo measurements. Note that setting this parameter too large hinders accurate field mapping. The resulting linear system was solved using the conjugate gradient (CG) method.

At the end of each iteration, the mapped $\Delta {B}_{0}$ field was fitted to a basis of up to second-order spherical harmonics before using it as input in the image reconstruction step. This was done to obtain an estimate of the field outside the object.

### Gradient nonlinearity correction

Gradient nonlinearity correction was furthermore combined with ${B}_{0}$ correction in iterative CPR and iterative MB using the gradient maps obtained from Biot–Savart simulations of the gradient fields in the commercial simulation package CST studio suite (Dassault Systèmes, Vélizy-Villacoublay, France). For CPR this was done in two steps. The first step corrects for nonlinearities of the readout gradient ${G}_{x}(\mathbf{x})$. Note that for each phase encoding step (modeled through different duration τ for a constant phase encoding gradient ${G}_{y}(\mathbf{x})$) Eq. (1) can be written as

7 $$y_{\tau}\left({t}\right)=\int {m}\left(\mathbf{x}\right){{\varvec{e}}}^{-2{\pi}\Delta {{B}}_{0}\left(\mathbf{x}\right)it}{e}^{-{2{\pi}G}_{{x}}\left(\mathbf{x}\right)it}{{e}}^{-{2{\pi}{G}}_{y}\left(\mathbf{x}\right){i}{\tau}}{d}\mathbf{x}.$$

The readout gradient was written as ${{G}_{x}(\mathbf{x})= G}_{x,\text{nonlinear}}(\mathbf{x})+{G}_{x,\text{linear}}(\mathbf{x})$ such that Eq. (7) turns into

8 $$y_\tau\left(t\right)=\int {m}\left(\mathbf{x}\right)e^{-2\pi\left(\Delta B_0\left(\mathbf{x}\right)+G_{x,\text{nonlinear}}\left(\mathbf{x}\right)\right)it}e^{-{2\pi G}_{x,\text{linear}}\left(\mathbf{x}\right)it}e^{-{2\pi G}_y\left(\mathbf{x}\right)i\tau}d\mathbf{x}.$$

Since the spatially nonlinear part of a readout gradient ${G}_{x,\mathrm{nonlinear}}$ presents itself in the same way as an inhomogeneous ${\Delta B}_{0}$ field in a spin-echo sequence, its effect on the reconstructed image was also corrected simultaneously with the ${\Delta B}_{0}$ field using the effective off-resonance map ${\omega }_{\mathrm{eff}}\left(\mathbf{x}\right)={\Delta B}_{0}\left(\mathbf{x}\right)+{G}_{x,\mathrm{nonlinear}}\left(\mathbf{x}\right)$ as input in CPR. Second, the nonlinearity of the phase encoding gradient was corrected in a similar way: the gradient was written as ${{G}_{y}(\mathbf{x})= G}_{y,\mathrm{nonlinear}}(\mathbf{x})+{G}_{y,\mathrm{linear}}(\mathbf{x})$ and the image $m$ in

9 $$y_\tau\left(t\right)=\int m\left(\mathbf{x}\right)e^{-2{\pi}G_{y,\text{nonlinear}}\left(\mathbf{x}\right)i\tau}e^{-2\pi G_{x,\text{linear}}\left(\mathbf{x}\right){i}{t}}{{e}}^{-2{\pi}G_{y,\text{linear}}\left(\mathbf{x}\right)i\tau}d\mathbf{x}$$

was approximated as

10 $$m\left(\mathbf{x}\right)=\int y_\tau\left(t\right) e^{2\pi G_{y,\text{nonlinear}} \left(\mathbf{x}\right) i \tau } e^{2\pi G_{x,\text{linear}}\left(\mathbf{x}\right)it} e^{2\pi G_{y,\text{linear}}\left(\mathbf{x}\right) i \tau} d \tau,$$

and efficiently calculated using a time map that is rotated by 90 degrees relative to the time map for the readout gradient. For MB, the gradient maps were directly incorporated into the system matrix $E$.

## Results

### Simulation experiments

**Fig. 2:**
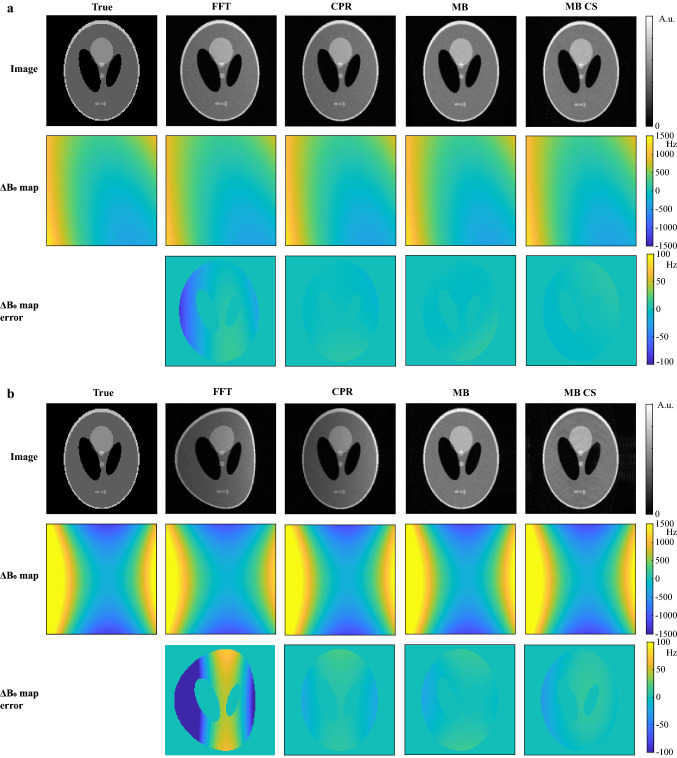
Comparison of FFT, iterative MB and iterative CPR approaches for simulation data in the center slice and in an off-center slice. **a** Center slice. (Top row) The FFT reconstruction results in a slight stretch of the Shepp–Logan phantom along the readout direction (left-to-right). (Middle row) The standard ${B}_{0}$ mapping technique (FFT) leads to inaccuracies in regions where the ${B}_{0}$ inhomogeneities are strongest (~ 600 Hz). The iterative CPR and MB approaches correct for this stretch using the estimated field map in each iteration and result in very similar image quality and $\Delta {B}_{0}$ maps. (Bottom row) This is confirmed by the low errors in the estimated $\Delta {B}_{0}$ maps. The MB CS reconstruction for an undersampling factor of 2 shows very similar errors to that of the MB approach. **b** Off-center slice. The stronger inhomogeneities in this slice (~ 1500 Hz) result in even larger errors using the standard ${B}_{0}$ mapping technique (FFT). Iteratively applying CPR and MB reduces the errors. For iterative MB image reconstruction, the uniformity of the image is closer to that of the true object compared to iterative CPR

Figure 2a shows a comparison between the different ${B}_{0}$ mapping and image reconstruction approaches for the simulated Shepp–Logan phantom in a slice in the center of the magnet. The $\Delta {B}_{0}$ map estimated using the FFT results in errors of 80 Hz compared with the $\Delta {B}_{0}$ map that was used to generate the simulated data and a slight image stretch is visible along the readout direction (left-to-right). Iterative CPR and iterative MB (5 iterations) both result in accurate $\Delta {B}_{0}$ maps (errors below 9 Hz) and reconstructed images with reduced distortion. Figure 2b shows the same comparison for a slice 7.5 cm away from the center of the magnet, where the main field inhomogeneities are much stronger. The $\Delta {B}_{0}$ map estimated using the FFT results in errors of 500 Hz. The estimated $\Delta {B}_{0}$ map for iterative CPR and iterative MB are both close to the true field map with maximum errors of 22 Hz. The reconstructed image using CPR, however, still shows left-to-right shading, while the MB approach results in a much more uniform signal intensity across the image. The MB CS reconstruction for an undersampling factor of 2 furthermore shows very similar errors in the $\Delta {B}_{0}$ map compared to that of the MB approach. The reconstructed images show some minor residual undersampling artifacts, which appear as ringing. In Online Resource 1 these simulations were repeated for a more complicated digital phantom [24], showing that MB CS can also recover fine image structures.

The computation times for one iteration of iterative CPR and one iteration of iterative MB reconstruction for a matrix size of 128 × 128 were 6.2 s and 21.6 s, respectively.

### Phantom and in vivo measurements

**Fig. 3:**
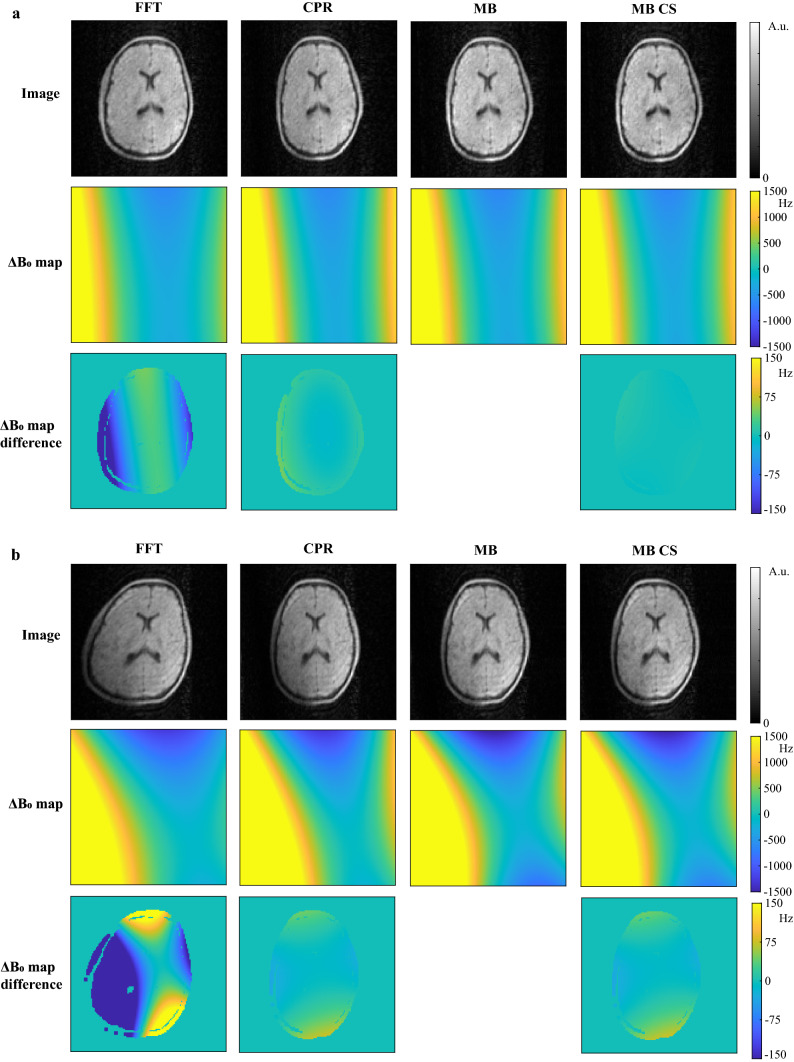
Comparison of FFT, iterative MB and iterative CPR approach for an in vivo brain scan. **a** Mild ${B}_{0}$ inhomogeneities (~ 600 Hz). (Top row) The FFT reconstruction results in a stretch of the brain along the readout direction (left-to-right). Both iterative CPR and iterative MB image reconstruction result in low errors in the estimated field map, and correct for the corresponding image deformations. The estimated $\Delta {B}_{0}$ maps (middle row) and the differences with respect to that of the FFT approach (bottom row) show a similar improvement in the accuracy of the field map estimation when using the iterative CPR and MB approaches. The MB CS reconstruction for an undersampling factor of 2 shows the flexibility of the MB approach towards undersampling. **b** For strong ${B}_{0}$ inhomogeneities (~ 1500 Hz), here obtained by attaching an external magnet to the Faraday cage, the iterative MB approach still results in a uniform signal intensity, while the iterative CPR approach shows more signal shading in the lower-left corner of the brain

Figure 3a shows a comparison of the three approaches for the in vivo brain scan in the center slice. The FFT reconstruction results in a stretch of the brain along the readout direction (left-to-right), similar to that produced in the simulations. For these mild ${B}_{0}$ inhomogeneities (~ 600 Hz), the iterative CPR and MB approach result in very similar image quality, which is in agreement with simulation results. The estimated $\Delta {B}_{0}$ maps and the differences with respect to that of the FFT approach, show an improvement in the accuracy of the field map estimation of approximately 200 Hz when using the iterative CPR and MB approaches. It is worth noting that the estimated field corresponds well with the measured field in Fig. 1c, which was measured for a slightly smaller FOV. The MB CS reconstruction with an undersampling factor of 2 furthermore shows very similar quality to that of the MB approach, demonstrating the flexibility of the MB approach towards undersampling. Figure 3b shows that while the accuracy of the field maps are comparable for CPR and MB (within 75 Hz) for stronger ${B}_{0}$ inhomogeneities (~ 1500 Hz), the iterative MB approach results in a more homogeneous signal intensity compared to the iterative CPR approach, which is in agreement with the simulation results in Fig. 2b.

**Fig. 4:**
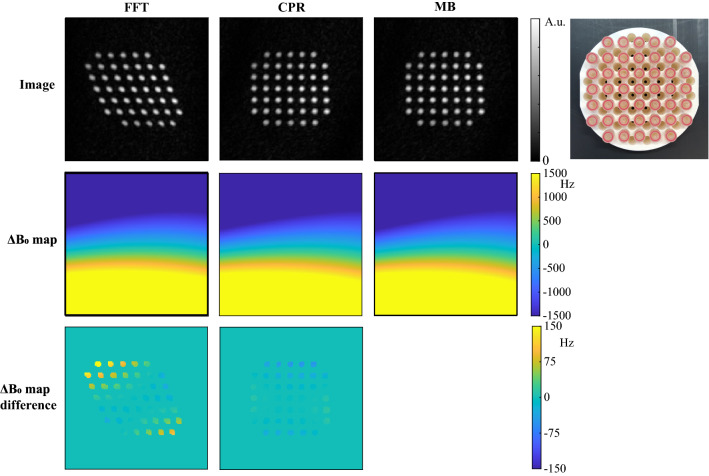
Comparison of FFT, iterative MB and iterative CPR approaches for strong (~ 1200 Hz) ${B}_{0}$ inhomogeneities in a grid phantom: 45 Equally spaced tubes filled with sunflower oil on a rectangular grid. Both MB and CPR result in images of which the structure is close to that of the true object. A photograph of the grid phantom is shown as a reference. Strong ${B}_{0}$ inhomogeneities were obtained by running a constant current through one of the gradient coils

To further test the robustness to large ${B}_{0}$ inhomogeneities, Fig. 4 shows ${B}_{0}$ mapping and reconstruction results for the phantom containing 45 equally spaced tubes filled with sunflower oil. Both CPR and MB result in corrections of approximately 150 Hz on the final $\Delta {B}_{0}$ map. The structure of the phantom in the reconstructed images using MB and CPR is close to that of the true object, of which a photograph is shown as a reference.

**Fig. 5:**
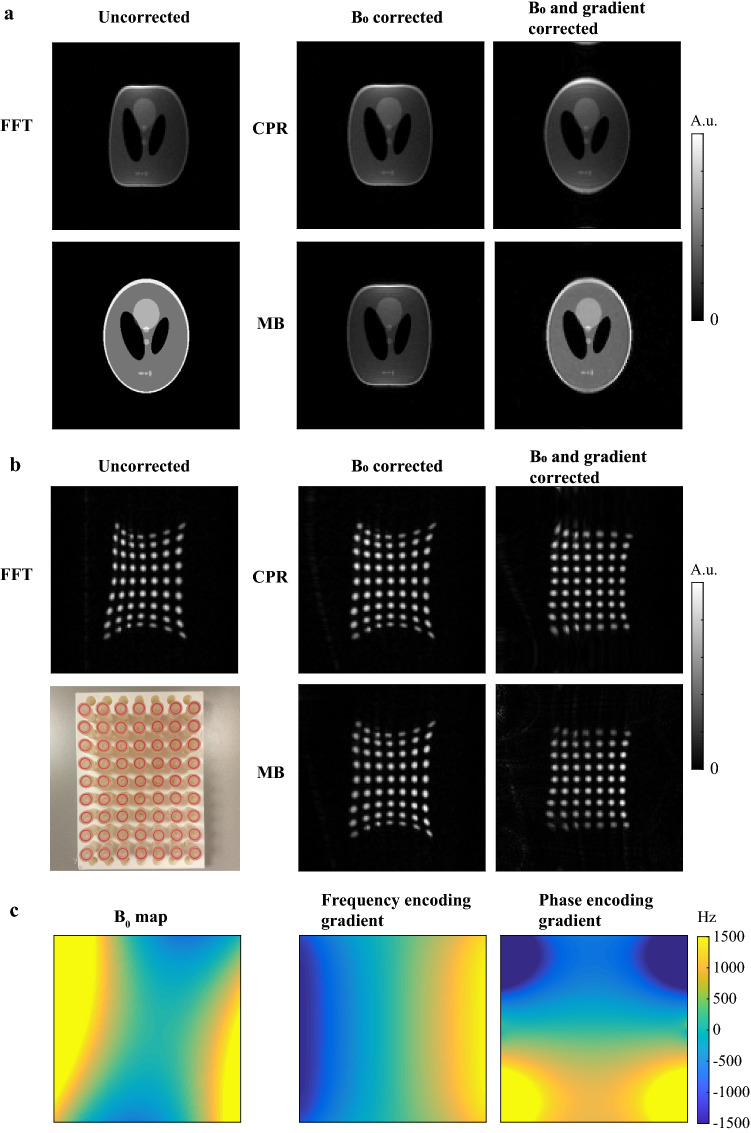
Combined gradient nonlinearity and ${B}_{0}$ correction for simulation and measurement data. **a** Gradient nonlinearities produce additional image distortions on top of ${B}_{0}$-induced image distortions for a simulated Shepp–Logan phantom when reconstructed with an FFT. Iterative CPR and iterative MB are able to correct for both type of distortions simultaneously, but iterative MB shows a more uniform signal intensity compared to iterative CPR. **b** Measurement data of a rectangular grid phantom, containing 63 equally spaced tubes filled with sunflower oil, show reduced ${B}_{0}$ and gradient distortions when reconstructed with iterative MB and CPR. Residual image distortions in measurement data are potentially caused by small differences between simulated gradient maps and actual gradient fields, which could, for example, arise when one of the gradient coils is slightly rotated with respect to its orthogonal position. A photograph of the phantom is shown in the lower-left corner. **c** The ${B}_{0}$ map was estimated from the measured data in (**b**), and subsequently used to simulate image distortions in (**a**). Maps of the frequency and phase encoding gradients were simulated in coronal orientation using Biot–Savart’s law

Figure 5 shows simulation and measurement data in a coronal orientation for which the gradient nonlinearity pattern is strong compared to that in the transverse orientations studied in Figs. 1, 2, 3, 4. When simulated gradient maps are taken into account in the reconstruction process, both iterative CPR and iterative MB show reduced image distortions compared to the FFT reconstruction and compared to the ${B}_{0}$ corrected images. Iterative MB shows a more uniform signal intensity compared to iterative CPR. Measurement data show some residual image distortions that could be explained by small differences between simulated gradient maps and actual gradient fields, which could arise when one of the gradient coils is slightly rotated with respect to its orthogonal position. Note that the inhomogeneities in the ${B}_{0}$ field now result in distortions in the opposite direction compared to in Figs. 2, 3, 4 due to the use of a reversed gradient direction.

## Discussion

The results in this paper showed that ${B}_{0}$ inhomogeneities present at the low field can be encoded into the measured k-space data using a TSE sequence with a time-shifted readout gradient and that their adverse effects on image quality can the mitigated by appropriate image reconstruction techniques. Standard Fourier transform-based ${B}_{0}$ mapping leads to inaccurate $\Delta {B}_{0}$ maps in the case of strong main field inhomogeneities. Iteratively applying CPR corrects for the image distortions and reduces the errors in the $\Delta {B}_{0}$ map, but in regions where the inhomogeneities are large compared to the gradient strength the images still suffer from reduced signal uniformity. Iteratively applying MB reconstruction results in higher image quality and in a similar ${\Delta B}_{0}$ error compared to iterative CPR. Combination of the MB approach with CS resulted in very similar image and ${\Delta B}_{0}$ map quality for an undersampling factor of 2 compared to the fully sampled iterative MB approach. This framework enables us to reconstruct ${B}_{0}$ artifact-free images without penalties on the total scanning time. This is particularly relevant for low field permanent magnet MRI systems, where main field inhomogeneities are often severe and may change in time, but could also be of interest in high field applications.

The experiments in this paper have shown that if gradient nonlinearities are known, either via simulation or via external measurement, they can easily be integrated into the forward model of MB or in CPR, and hence support ${B}_{0}$ inhomogeneity and spatial gradient nonlinearity correction simultaneously. This can be done as long as the gradients produce a unique gradient encoding for each location. In future work, the MB model could be extended to larger ${\Delta B}_{0}$ inhomogeneities by incorporating a frequency range within each voxel in the encoding matrix instead of a single frequency [25, 26]. Furthermore, incorporating local field changes within each voxel via Taylor series expansion of the ${\Delta B}_{0}$ map, as done in [27], can improve the accuracy of the signal model further.

In this work, we used the ${\ell}_{1}$-norm TV operator acting on the image as regularization in the image reconstruction step. Its sparsifying effect becomes especially important when MB reconstruction is combined with CS. There are other regularization operators that could be used instead, potentially creating sparser representations than TV, hence allowing larger undersampling factors. A different type of regularization was needed for the field map estimation step since neither the field itself nor its derivative is sparse. We enforced the inherent smooth structure of the field by minimizing jumps in the field via the $\ell_{2}$-norm TV operator acting on the field map. As an alternative approach one could also think about enforcing sparsity of the coefficients of the spherical harmonics decomposition of the field. However, enforcing smoothness of the field through $\ell_{2}$-norm minimization is more straightforward and much less computationally expensive compared to $\ell_{1}$-norm minimization.

Field drift due to temperature changes is in general a well-known problem in low field permanent magnet MRI systems [4]. In our experiments, we have not observed large field drifts. However, if temperature changes are tracked during acquisition, field drifts can be modelled and considered in the forward model of the MB reconstruction. Part of this problem can also be mitigated by shortening the scan times using k-space undersampling and CS, for which the current MB reconstruction framework was shown to be particularly suitable as it already incorporates a total variation transform for regularization. Reconstructed $\Delta {B}_{0}$ maps could also be used to correct other $\Delta {B}_{0}$ distorted images from the same scan session.

Another class of approaches estimates a $\Delta {B}_{0}$ map from k-space data before it is used in an MB image reconstruction step [17, 21]. The advantage of such approaches is that the field map is encoded along the entire readout duration of each time-domain signal, whereas for image-based ${B}_{0}$ mapping the field map is encoded in the phase for each pixel separately. This means that, while image-based ${B}_{0}$ mapping needs at least two acquisitions, time-domain approaches potentially need one acquisition [21], hence can lead to a shorter acquisition time. A downside, however, is that time-domain approaches require longer reconstruction times compared to image-domain approaches due to the need to construct and use a large dense matrix for the field map error estimation step [17, 21]. Furthermore, while accurate at clinical field strengths [17], the linearization step that is needed to derive a linear system for the error estimation step will become inaccurate for the strong inhomogeneities that we are dealing with at low field (${\Delta B}_{0}\approx$ 3000 Hz) [21]. For these reasons, we expect that such approaches will fail to fully correct for all ${B}_{0}$-induced image distortions for our current magnet. Further research is needed to compare the accuracy of image-based ${B}_{0}$ mapping and time-domain field map estimation and their effects on the MB reconstructed images.

Although iterative MB reconstruction is able to produce a more accurate image intensity for stronger inhomogeneities than iterative CPR, it also has a longer computation time than iterative CPR. This is explained by the fact that CPR can be efficiently implemented using FFTs. In case of moderate inhomogeneities for which CPR provides sufficient accuracy, iterative CPR would therefore offer a more practicable solution than iterative MB reconstruction. Also hybrid approaches, in which CPR and MB are combined, could be useful for these cases. It should be noted, however, that reconstruction codes used for the comparisons performed in this paper have not been fully optimized for speed. Code optimization will be necessary to obtain clinically acceptable reconstruction times for 3D or multi-slice reconstructions. Currently, the two most expensive steps in each iteration of the proposed approach are updating the SB system matrix and reconstructing the two images. These steps can be sped up considerably with the help of parallelization. Further reduction in computation time can be achieved by using preconditioning techniques [13, 28, 29], although this would only have a significant impact for extremely strong inhomogeneity cases, where the structure of the system matrix is very different from that of the Fourier transform.

In conclusion, both iterative CPR and iterative MB can help to reduce ${B}_{0}$ distortions in reconstructed images and improve the accuracy of ${B}_{0}$ mapping for a strongly inhomogeneous main field, although iterative MB generally results in a higher signal uniformity across the imaging object than CPR.

## Supplementary Information

Below is the link to the electronic supplementary material.

Online Resource 1 Comparison of FFT, iterative MB and iterative CPR approaches for a digital CS phantom. The FFT reconstruction results in a stretch of the phantom along the readout direction (left-to-right) in regions where the *B*_0_ inhomogeneities are strongest (~800 Hz). The iterative CPR and MB approaches correct for this stretch using the estimated field map in each iteration and result in very similar image quality. (Bottom row) This is confirmed by the low errors in the estimated ∆*B*_0_ maps. The MB CS reconstruction for an undersampling factor of 2 shows very similar errors to that of the MB approach. All approaches show a fold-over artifact in the left bottom corner, where the distorted signal is pushed outside the FOV due to the strong inhomogeneities. (EPS 3919 KB)
